# Dupilumab-Associated Eosinophilic Granulomatosis With Polyangiitis

**DOI:** 10.7759/cureus.27670

**Published:** 2022-08-04

**Authors:** Purnadeo Persaud, Rehan Karmali, Parvathy Sankar, Muhammad Majid

**Affiliations:** 1 Internal Medicine, Cleveland Clinic, Cleveland, USA; 2 Cardiology, Cleveland Clinic, Cleveland, USA

**Keywords:** eosinophilic granulomatosis with polyangiitis, dupilumab, human igg4 monoclonal antibody, atopic disease, chronic sinusitis

## Abstract

A 58-year-old Caucasian male developed generalized weakness, bilateral upper and lower extremity arthralgias, left pedal edema, and a new rash over two weeks after receiving one dose of dupilumab for chronic sinusitis. He was found to be positive for perinuclear anti-neutrophil cytoplasmic antibodies and myeloperoxidase, with evidence of interstitial lung disease on CT imaging. A skin biopsy showed eosinophilic vasculitis and a kidney biopsy showed pauci-immune glomerulonephritis. He was diagnosed with eosinophilic granulomatosis with polyangiitis due to dupilumab exposure.

## Introduction

Dupilumab is a fully human IgG4 monoclonal antibody against the interleukin (IL)-4 receptor-alpha subunit, inhibiting the signaling of IL-4 and IL-13. It was developed as a potential agent for the treatment of atopic or allergic diseases [1]. In March 2017, dupilumab became the first targeted biologic therapy that was globally approved for the treatment of atopic dermatitis [1]. Since then, it has also been used for the treatment of eosinophilic asthma, and chronic rhinosinusitis associated with nasal polyps [2]. Current investigations are underway for its use in eosinophilic esophagitis and chronic obstructive pulmonary disease [2,3]. Common adverse effects from this medication include local site reactions, nasopharyngitis, and conjunctivitis. More rarely, there have been case reports of alopecia areata, facial rashes, and thyroiditis [4]. Here we report a rare case of eosinophilic granulomatosis with polyangiitis (EGPA) with joint, skin, lung, and renal involvement shortly after beginning therapy with dupilumab.

## Case presentation

Initial presentation

A 58-year-old Caucasian male with a past medical history significant for a severely deviated septum with chronic sinusitis, coronary artery disease, hypertension, hyperlipidemia, and well-controlled type 2 diabetes presented to the emergency department (ED) with two weeks of generalized weakness, bilateral upper and lower extremity arthralgias, left pedal edema, and new rash. The patient had been suffering from chronic sinusitis for about three years associated with congestion, post-nasal drip, and cough. After unsuccessful treatment with antihistamines and inhaled and short-term use of oral glucocorticoids, he received a dose of dupilumab in the outpatient setting. He reported that his presenting symptoms began approximately a week after this treatment, first as a rash of his elbows, then arthralgias and weakness, followed by further rashes. A review of symptoms was otherwise negative for fever or chills, unexpected weight change, chest pain, dry eyes, shortness of breath, or any urinary symptoms. He is a former smoker of seven pack-years who quit three years prior and had no history of illicit drug use. Family history was not significant for any pulmonary, autoimmune, or kidney disease.

**Figure 1:**
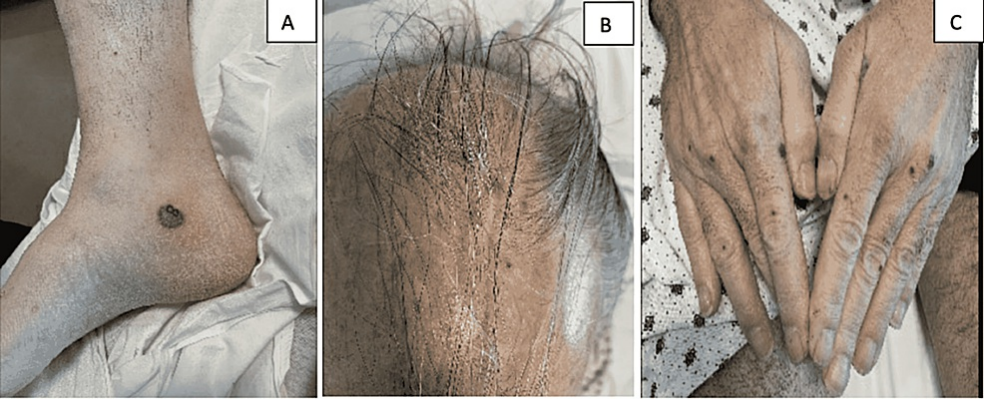
Skin findings (A) Violaceous bullae of the right ankle. (B) Macules of the scalp. (C) Raised violaceous lesions on the dorsal side of the hands.

Upon arrival to the ED, he was noted to be hypertensive with a blood pressure of 158/91 mmHg and tachycardic with a heart rate of 103 beats per minute. Physical examination was notable for <1 cm non-tender circular violaceous bullae and macules of the right medial ankle, the dorsal side of both hands, fingertips, and posterior scalp (Figure 1). There was no surrounding erythema or warmth, sloughing, or fluctuance associated with the skin lesions. He also had 1+ edema of the left foot with trace edema of the right. Initial labs were significant for leukocytosis (31.7 k/uL) with eosinophilia (70.7%), mild anemia (hemoglobin 11.5 g/dL), elevated erythrocyte sedimentation rate (88 mm/h) and C-reactive protein (54 mg/dL), hyperkalemia (5.6 mmol/L), elevated creatinine (2.98 mg/dL) and blood urea nitrogen (37 mg/dL), and elevated alkaline phosphatase (255 U/L) and aspartate aminotransferase (52 U/L). Prior labs three years ago did not have leukocytosis and elevated transaminases, and baseline creatinine was approximately 0.9 mg/dL. Chest X-ray was negative for any acute process but had signs of suspected chronic interstitial lung changes. An X-ray of his left ankle showed only soft tissue swelling of the fifth digit, but no fracture or sign of infectious process.

Given the onset of symptoms shortly after his first dupilumab injection along with eosinophilia, arthralgia, skin rash, and serum sickness-like reaction, suspicion was high for an adverse drug reaction. Infectious etiology was deemed less likely given the absence of abscess-like purulent drainage, cellulitic findings from his rash, absence of fever, and imaging not concerning for cellulitis/osteomyelitis. Given the new acute kidney injury along with systemic symptoms and eosinophilia, inflammatory and vasculitic causes such as EGPA, mixed cryoglobulinemia (given elevated liver enzymes), and malignancy such as lymphoma or chronic myeloid leukemia were considered as part of the differential diagnoses.

Hospital course and management

**Figure 2:**
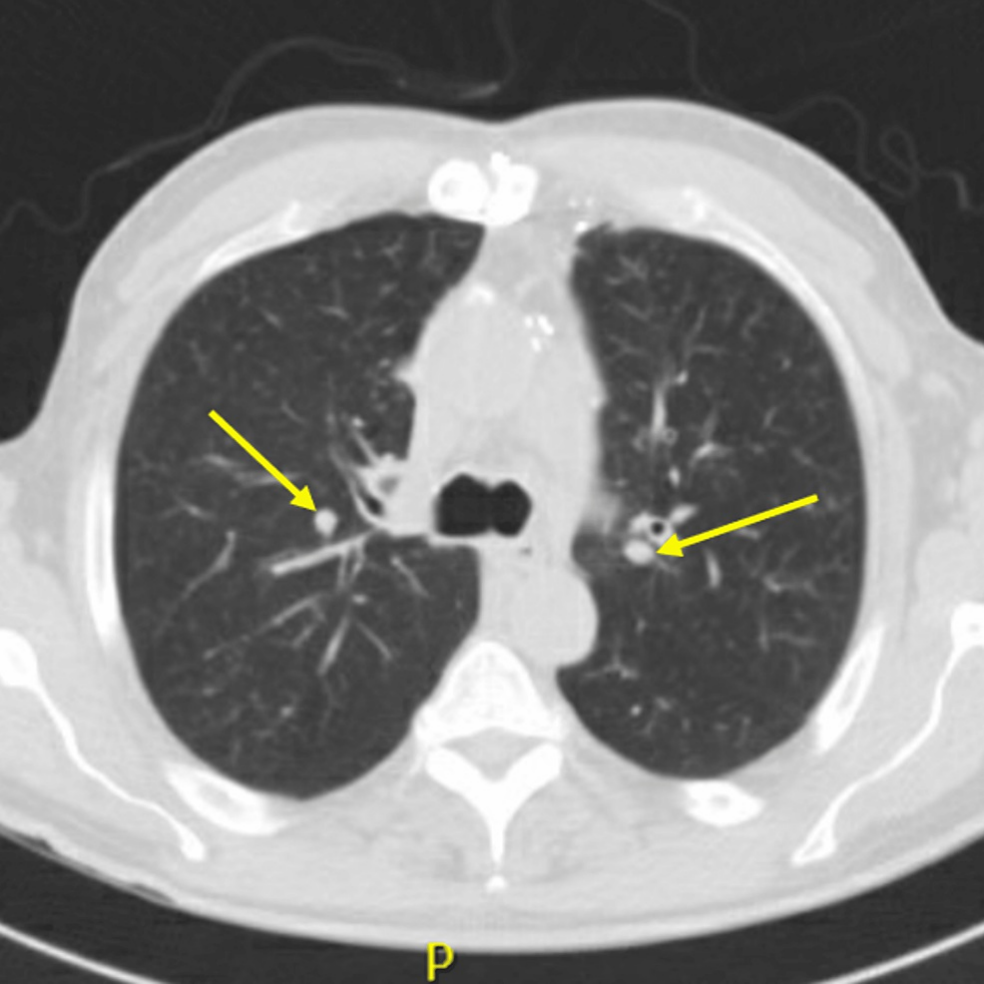
Chest CT findings Clustered centrilobular nodular opacities bilaterally and multiple new pulmonary nodules indicated by yellow arrows.

**Figure 3:**
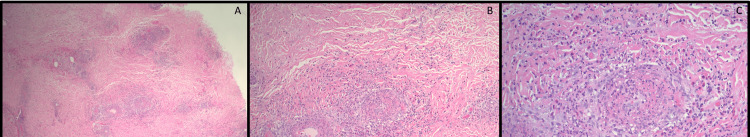
Skin biopsy findings Skin biopsy showing hyperkeratosis overlying a necrotic epidermis with festooning of the dermal papillae. Dense perivascular inflammatory infiltrate composed of neutrophils and numerous eosinophils with fibrinoid necrosis of the vessel walls, karyorrhexis, and extravasated erythrocytes. Findings suggestive of vasculitis and vasculopathy, specifically EGPA low power (A), 10x (B), and 20x magnification (C). EGPA, eosinophilic granulomatosis with polyangiitis.

**Figure 4:**
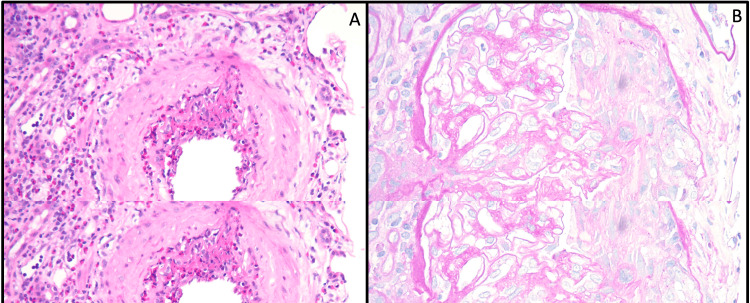
Renal biopsy findings (A) Glomerulus with a necrotizing cellular crescent (PAS stain, 400x magnification). (B) Glomerulus with necrotizing arteritis with eosinophil-rich inflammation (H&E, 200x magnification). PAS, periodic acid-Schiff; H&E, hematoxylin and eosin.

Due to the patient’s presenting symptomology and labs, dermatology, rheumatology, and nephrology were consulted. The patient was found to be positive for perinuclear anti-neutrophil cytoplasmic antibodies (p-ANCA) and myeloperoxidase. He was negative for antinuclear antibodies, and complement components were within normal limits. the hepatitis panel was negative, and ultrasound ruled out deep vein thrombosis in the lower extremities. An echocardiogram of the heart was negative for vegetation ruling out emboli. Urine studies showed dysmorphic red blood cells and granular muddy brown casts. CT chest was done and was compared to the last CT three years ago. It showed increased diffuse bronchial wall thickening, diffuse clustered centrilobular nodular opacities bilaterally, and multiple new pulmonary nodules <6 mm likely infectious or inflammatory in etiology (Figure 2). A skin biopsy of the rash was also done and showed vasculitis and vasculopathy with numerous eosinophils consistent with eosinophilic vasculitis (Figure 3). Furthermore, a renal biopsy was done, which showed pauci-immune glomerulonephritis (Figure 4). 

The combination of the above findings was consistent with eosinophilic granulomatosis polyangiitis with joint, skin, lung, and renal involvement. For treatment of this condition, he received a course of methylprednisolone for three days with a transition to oral prednisone, with a plan for long-term rituximab beginning in the outpatient setting. During his stay, leukocytosis and eosinophilia improved back to baseline levels. His rash also improved, and his creatinine and alkaline phosphatase down trended. On follow-up approximately one month later, the patient had decreased rash without any new complaints.

## Discussion

EGPA is the rarest among ANCA-associated vasculitis and has an incidence of 0.9-2.4 per million people [5]. To the best of our knowledge, this is one of the very few cases of EGPA in patients after receiving dupilumab. Two multicenter, double-blind phase 3 trials were done on several hundred patients and found that only two patients were diagnosed with EGPA after using the drug [6]. One of the two individuals was diagnosed 300 days after a single dose. The other was a 41-year-old male who developed ANCA-negative EGPA after a sudden stoppage of steroids before starting dupilumab [7]. Our patient did receive steroids for chronic sinusitis in the past, but it was only a five-day course of 20 mg prednisone four months prior. These findings suggest that EGPA may not only arise in patients who had masked EGPA while on steroids, but it can also occur from a direct drug-related process. On the contrary, a case by Adachi et al. showed that treatment with dupilumab reduced symptoms caused by EGPA such as leg edema, abdominal pain, and dyspnea [7]. Another case by Ikeda et al. included a patient who presented with EGPA only after discontinuing dupilumab [8].

In our patient, we observed leukocytosis and eosinophilia. Lommatzsch et al. presented a case that involved dupilumab-induced hypereosinophilia, which led to eosinophilic pleural effusion within the first six weeks after treatment initiation [9]. Another study showed that blood eosinophils transiently increased after dupilumab in patients with asthma or chronic sinusitis, but not in atopic dermatitis or eosinophilic esophagitis. The eosinophilia generally declined to below baseline levels over time [6]. Based on these sources, it appears that eosinophilia could be expected with early medication use like our patient. 

In summary, after comprehensive literature review, our patient’s symptoms could be explained by three possible causes. First, he may have had underlying EGPA that was unmasked by dupilumab. Alternatively, dupilumab may have directly induced his EGPA. The final one is the possibility that the patient had EGPA that coincidentally started after dupilumab use.

## Conclusions

Dupilumab is the first biologic that targets TH2-mediated allergic diseases. It has proven its beneficial effects in the treatment of moderate-severe atopic dermatitis and eosinophilic asthma. Rare side effects to be mindful of dupilumab include serum-like sickness, facial rashes, and thyroiditis, among other adverse effects. The association of EGPA with dupilumab remains unclear and it is imperative to monitor patients starting this medication. Further investigations and case reports are needed to strengthen the association and the underlying mechanism.

## References

[1] Shirley M (2017). Dupilumab: first global approval. Drugs.

[2] Matsunaga K, Katoh N, Fujieda S, Izuhara K, Oishi K (2020). Dupilumab: basic aspects and applications to allergic diseases. Allergol Int.

[3] Hirano I, Dellon ES, Hamilton JD (2020). Efficacy of dupilumab in a phase 2 randomized trial of adults with active eosinophilic esophagitis. Gastroenterology.

[4] Albader SS, Alharbi AA, Alenezi RF, Alsaif FM (2019). Dupilumab side effect in a patient with atopic dermatitis: a case report study. Biologics.

[5] Furuta S, Iwamoto T, Nakajima H (2019). Update on eosinophilic granulomatosis with polyangiitis. Allergol Int.

[6] Wechsler ME, Klion AD, Paggiaro P (2022). Effect of dupilumab on blood eosinophil counts in patients with asthma, chronic rhinosinusitis with nasal polyps, atopic dermatitis, or eosinophilic esophagitis. J Allergy Clin Immunol Pract.

[7] Murag S, Melehani J, Filsoof D, Nadeau K, Chinthrajah RS (2021). Dupilumab unmasks eosinophilic granulomatosis with polyangitis. Chest.

[8] Ikeda M, Ohshima N, Kawashima M, Shiina M, Kitani M, Suzukawa M (2022). Severe asthma where eosinophilic granulomatosis with polyangiitis became apparent after the discontinuation of dupilumab. Intern Med.

[9] Lommatzsch M, Stoll P, Winkler J, Zeise-Wehry D, Tronnier M, Weber MA, Virchow JC (2021). Eosinophilic pleural effusion and stroke with cutaneous vasculitis: two cases of dupilumab-induced hypereosinophilia. Allergy.

